# 640-Gbit/s fast physical random number generation using a broadband chaotic semiconductor laser

**DOI:** 10.1038/srep45900

**Published:** 2017-04-04

**Authors:** Limeng Zhang, Biwei Pan, Guangcan Chen, Lu Guo, Dan Lu, Lingjuan Zhao, Wei Wang

**Affiliations:** 1 Key Laboratory of Semiconductor Materials Science, Institute of Semiconductors, Chinese Academy of Sciences, Beijing Key Laboratory of Low Dimensional Semiconductor Materials and Devices, Beijing, 100083, China

## Abstract

An ultra-fast physical random number generator is demonstrated utilizing a photonic integrated device based broadband chaotic source with a simple post data processing method. The compact chaotic source is implemented by using a monolithic integrated dual-mode amplified feedback laser (AFL) with self-injection, where a robust chaotic signal with RF frequency coverage of above 50 GHz and flatness of ±3.6 dB is generated. By using 4-least significant bits (LSBs) retaining from the 8-bit digitization of the chaotic waveform, random sequences with a bit-rate up to 640 Gbit/s (160 GS/s × 4 bits) are realized. The generated random bits have passed each of the fifteen NIST statistics tests (NIST SP800-22), indicating its randomness for practical applications.

High-quality random numbers are the key component of multiple technologies, such as secure communicatioτns^1^, quantum communications^2^, Monte-Carlo simulations^3^, stochastic modeling^4^ and even lotteries. The unpredictability and irreproducibility of the random bits are of paramount importance in those applications. Moreover, the generation speed of the random bits determines the bit-rate of secure communication and data encryption. It is necessary to realize high-speed random numbers to keep up with the data rate of modern communication systems.

Random numbers generated by deterministic algorithms have been commonly used in many systems^5^,^6^. It adds little hardware cost, but the speed is limited by the processing chips and its deterministic and periodical nature will cause serious problems, such as data theft in communication systems and fatal mistakes in computing. Physical random numbers generated by intrinsically non-deterministic physical processes can overcome those flaws and ensure the confidentiality. The commonly used physical processes contain thermal noise in resistors^7^, frequency jitter of oscillators^8^, amplified spontaneous emissions from super-luminescent diodes^9^, the spatial resolution of single photon emission^10^ and photon events in attenuated light^11^. However, their bit rates are limited by the bandwidths of physical entropy sources, which are generally around or below GHz scale.

With the advantages in intensity and bandwidth, optical chaos signal generated by laser diode has been proved to be an excellent entropy source for physical random number generation (RNG). It consists of ultra-short pulses with irregular amplitudes and phases under nanosecond scale. However, due to the intrinsic relaxation oscillation or required external feedback, the generated signal contains non-eliminated correlations^12^. Therefore, post data processing is indispensable to enhance the randomness of chaos-based RNG.

In 2008, Uchida *et al*.^13^ have reported the first chaotic laser based RNG, showing the feasibility and potential of this technology. They have used exclusive-OR (XOR) of two chaotic signals with different feedback delays to suppress the time delay signature and a RNG rate of 1.7-Gbit/s has been achieved. Since then, many efforts have been devoted to increase the bit-rate of chaos-based RNG. One of the promising methods is the optimization of post data processing. In 2009, Reidler *et al*.^14^ have proposed a novel least significant bits (LSBs) interception method, which multiplies the generation rate through multiple-bit sampling. They have enhanced the RNG rate to 12.5 Gbit/s by using 5-LSBs sampled at 2.5 GS/s. At the same year, Kanter *et al*.^15^ have further increased the RNG rate to 300 Gbit/s through calculating high-order derivative of the chaotic waveform and retaining a number of LSBs. Based on this approach, in 2014, Li *et al*.^16^ have successfully achieved a 2.2 Tbit/s random bits by calculating 62th-order finite differences with 55 LSBs retaining. And in 2016, Butler *et al*.^17^ have realized a 1 Tbit/s generation rate using 10 retained valid bits after calculating 7th-order derivatives. In 2012, Akizawa *et al*.^18^ have proposed and utilized a bit-order-reversal method, where 400-Gbit/s random bits have been realized. In 2015, Tang *et al*.^19^ have realized a 1.12 Tbit/s RNG rate by merging of two random-bit streams processed by the bit-revise and LSBs methods. Another way for fast RNG is enhancing the bandwidth of the chaotic source. In 2010, Hirano *et al*.^20^ have achieved a 75-Gbit/s RNG rate by utilizing a bandwidth-enhanced chaotic laser, where a cascading optical feedback and injection structure was used to realize a 16-GHz chaotic bandwidth. In 2015, Sakuraba *et al*.^21^ have further increased the RNG rate to 1.2 Tbit/s by using a 35.2-GHz bandwidth-enhanced three-cascaded laser structure. In addition, several photonic integrated chaotic entropy sources^22^,^23^,^24^,^25^ have been developed to improve the robustness and compactness of the RNG system. Those devices have shown high performance, but their chaotic bandwidths were around or below 10 GHz, which resulted in a relatively lower RNG rate.

By optimizing the post data processing method and enhancing the chaotic bandwidth, the chaos-based RNG rate has already exceeded Tbit/s^16^,^17^,^19^,^21^. However, in order to enhance the RNG rate, merging of two random-bit streams processed by bit-revise and LSBs methods were used in post data processing^19^,^21^ or only “quasi-physical” random bits could be generated since high-order derivative method was needed to acquire additional valid bits exceeded the raw ADC^16^,^17^. These complicated post data processing methods could hardly work on-line since high-speed computing and high-frequency clock synchronization circuits were indispensable. Enhancing the chaotic bandwidth is a promising way to realize high RNG rate with simple post data processing method. However, currently developed bandwidth enhanced chaotic sources were complex, need precise control of the system and the enhanced bandwidth was still limited^20^,^21^. Thus, a simple and compact chaotic source with much wider bandwidth would be very attractive. Furthermore, a simple post data processing method is critically important to weak algorithm effect for high quality physical RNG and to simplify the electronic back ends in practical applications.

In this work, a compact and high-bit-rate RNG scheme is demonstrated using a PIC-based broadband chaotic source with a simple post data processing. The chaotic signal is generated by a monolithic integrated dual-mode amplified feedback laser (AFL) with self-injection, which has an RF frequency range of above 50 GHz and a spectrum flatness of within ±3.6 dB. The chaotic intensity is converted into an 8-bit digital signal by sampling with a wideband digital oscilloscope at 160 GS/s. After 4-least significant bits (LSBs) truncation, 640-Gbit/s physical random numbers are successfully generated and passed all 15 NIST statistics tests (NIST SP800-22). This high-quality and high-bit-rate random number generator is compact, simple and easy for fully integration, which can be a reliable technique for future communication and computing systems.

## Experimental Setup

**Figure 1:**
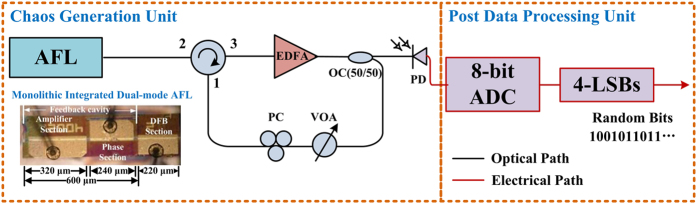
The schematic diagram of fast physical random number generation setup. Inset: microscope photography of the monolithic integrated dual-mode amplified feedback laser (AFL). EDFA, erbium-doped fiber amplifier; OC, optical coupler; VOA, variable optical attenuator; PC, polarization controller; PD, photodetector; ADC, analog-to-digital converter; LSBs, least significant bits.

Figure 1 shows the schematic diagram of the fast physical RNG setup. The broadband entropy source is originated from an optical feedback system employing a monolithic integrated dual-mode amplified feedback laser (AFL) as the light source. The post data processing uses only the simple LSBs method.

### Chaos generation unit

The dual-mode AFL, as shown in the inset of Fig. 1, is formed by a DFB laser section with a short integrated feedback cavity. The 220-*μ*m DFB laser section functions as the laser source and the integrated feedback cavity consists of a 240-*μ*m phase section and a 320-*μ*m amplifier section allowing the control of the feedback phase and strength. By adjusting the DC bias currents of each section, the device can work in the dual-mode state as long as the two laser modes have comparable threshold gain. Detailed fabrication and characterization of this device have been reported in our early paper^26^. The fiber-based external optical feedback loop is used to drive the two lasing modes entering the chaos state. Under proper mode-spacing, nonlinear coupling of the two chaotic modes will generate a broadband chaotic signal with a flat RF spectrum. Compared with traditional optical feedback systems using single-mode-lasers, the chaotic bandwidth, as well as spectrum flatness of the proposed scheme enhanced greatly with no additional system complexity.

In the measurement, the laser was mounted in butterfly package with a fiber pigtail coupled from the facet of the DFB section. It employed no optical isolator to allow external optical feedback. The temperature of the device was maintained at 25 °C by a thermoelectric cooler (TEC). An optical circulator was used to guide the output of the laser feedback to itself. The fiber feedback loop consisted of an erbium-doped fiber amplifier (EDFA, Amonics IL-13) and a variable optical attenuator (VOA), which boosted the output power and controlled the feedback strength, respectively. A polarization controller (PC) was used to adjust the polarization. It could be omitted if polarization maintaining fibers were used for all the fiber components. The fiber length of the feedback loop was 22.5 m, corresponding to a feedback delay time (round-trip) of 220 ns. This relatively long delay time is resulted from the long fiber loop. It could be efficiently reduced either when a SOA is employed to replace the EDFA or by shortening the pigtail of fiber devices.

### Post data processing unit

The generated chaos signal was converted into an electronic signal through a broadband photodiodes (*u*^2^t XPDV2320R, 50 GHz bandwidth), and then acquired by a wideband digital oscilloscope (Keysight Z-594d, 59-GHz analog bandwidth, 160 GS/s sample rate, 8-bit vertical resolution). The signal was also monitored by an RF spectrum analyzer (ESA, Agilent PXA N9030A, 50-GHz bandwidth) and an optical spectrum analyzer (Advantest Q8384, 0.01 nm resolution).

In the post data processing stage, a LSBs-only method is used to eliminate the time delay signature and enhance the randomness of the signal. This simple method corresponds to a relatively simpler electronic back ends and more easily to be implemented in real applications. In the experiments, the chaotic waveform was digitized by an 8-bit analog-to-digital converter (ADC) in the oscilloscope. Random bit is then generated after retaining a number of LSBs by a computer.

## Experimental Results

### Property of PIC-based chaotic source

**Figure 2:**
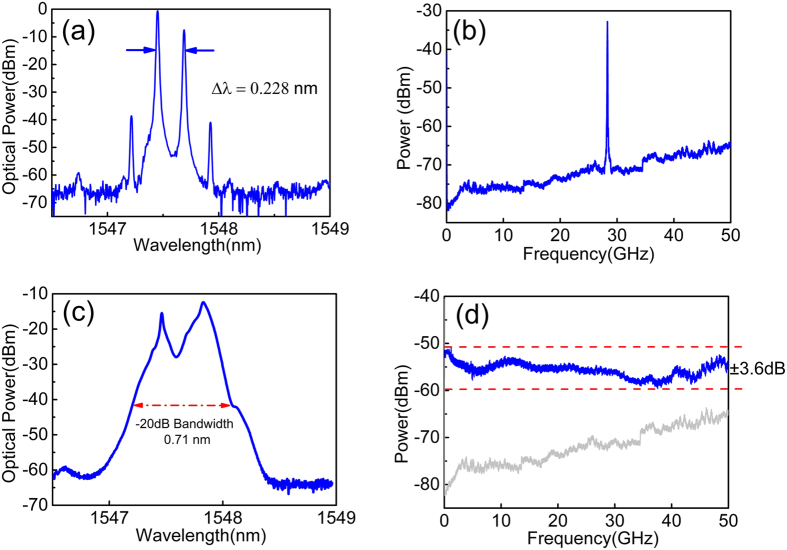
Measured (**a**) optical and (**b**) RF spectra of the free running dual-mode AFL; measured (**c**) optical and (**d**) RF spectra of the chaotic AFL after optical feedback.

**Figure 3:**
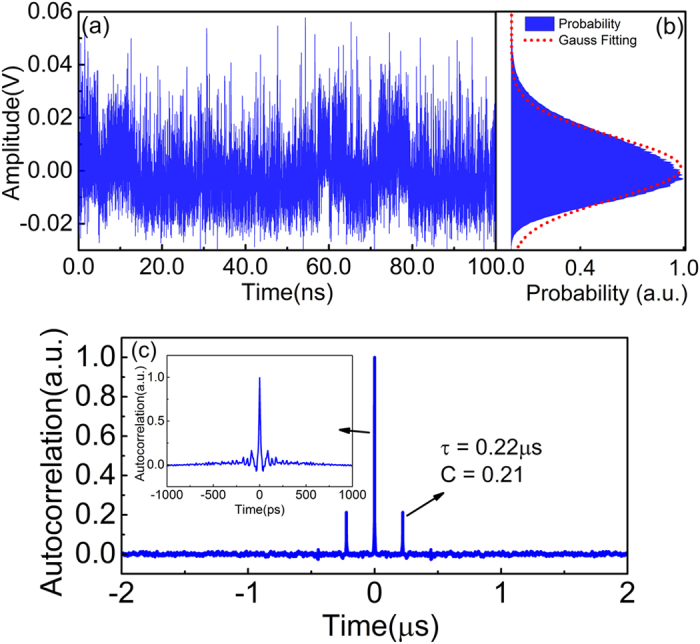
Measured (**a**) time trace, (**b**) probability density distribution and (**c**) autocorrelation function of the generated broadband chaotic waveform.

Firstly, the property of the broadband chaotic source is investigated. When the bias currents of DFB section (*I*_*DFB*_), phase section (*I*_*P*_), and amplifier section (*I*_*A*_) were 80 mA, 0 mA and 70 mA, respectively, the AFL worked in the dual-mode state as the optical spectrum shown in Fig. 2(a). The mode spacing of the two wavelengths was 0.228 nm, beating of which at a PD generated a 28.4-GHz RF signal as shown in Fig. 2(b). By adjusting the DC currents of each laser section, beating frequency of the device could be tuned continuously from 25 to 41 GHz, which greatly facilitates the choosing of optimal lasing conditions in chaos generation. When the feedback loop is connected, both the power feedback to the dual-mode AFL and delivered to the PD can be controlled through the EDFA and VOA. By setting the injection power as 6 dBm, which was estimated from the power at port 1 of the circulator, the two lasing modes entered chaotic state simultaneously. Feedback strength (*η*) of this state is 0.15, which is defined as the power ratio of the feedback signal and laser output in consideration of the coupling loss at laser facet (≈50%). Through the nonlinear coupling of the two chaotic wavelengths in the laser cavity, a broadened optical spectrum is achieved as shown in Fig. 2(c). The −20-dB bandwidth of the spectrum is 0.71 nm. The corresponding RF spectrum is illustrated in Fig. 2(d), which shows a flat distribution and covers an RF frequency range up to the frequency limit (50 GHz) of the ESA. The flatness of this spectrum is ±3.6 dB, which is the difference between the maximum and minimum powers in the whole frequency range. Figure 3(a) presents the temporal waveform of the chaotic signal, which shows noise-like intensity oscillation in a sub-nanosecond scale. The calculated probability density distribution of the temporal waveform is plotted in Fig. 3(b). The asymmetry of the distribution is identified by comparing it with the Gaussian fitted curve, which is plotted as the red dotted line in 3(b). As can be seen, the original chaotic intensity deviates from a Gaussian distribution. The autocorrelation trace is shown in Fig. 3(c). It can be clearly seen that peaks appear at the delay time (*τ*) of the external feedback loop, which is located at 0.22 *μ*s with a correlation coefficient (C) of 0.21. The inset of Fig. 3(c) is the zoom-in view of the center autocorrelation peak. As can be seen, a series of side lobes around the main peak appear, which are caused by the relaxation oscillation of the laser. Those peaks caused by intrinsic laser characteristics and delay feedback structure would induce periodicity and have bad influences on random number generation. Post data processing is used to eliminate them.

**Figure 4:**
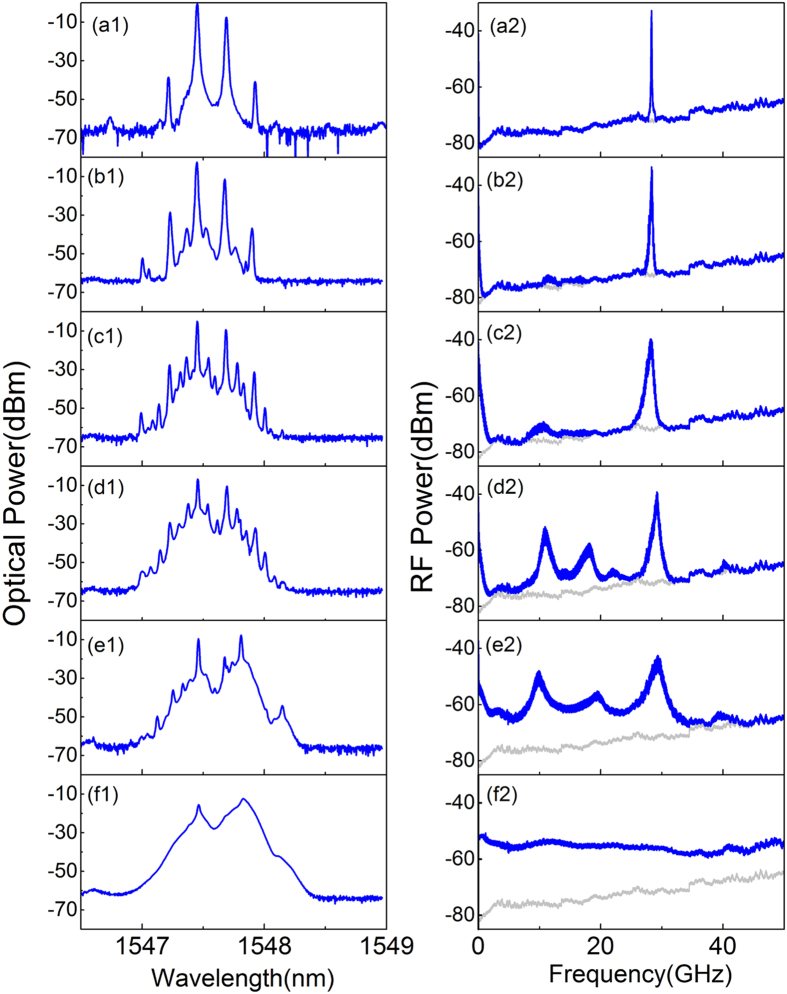
Dynamic routes into the chaotic state of the dual-mode AFL with optical feedback. Feedback strength from the top to the bottom are (**a**) free-running; (**b**) −5 dBm; (**c**) −2.9 dBm; (**d**) −0.8 dBm; (**e**) 3.7 dBm and (**f**) 6 dBm. The first and second column illustrate the optical spectrum and RF spectrum (RBW: 3 MHz; VBW: 1 kHz), respectively. The gray lines in RF spectra are the noise floor.

To further investigate this chaotic source, the dynamic processes of the dual-mode AFL evolving into the chaotic state are presented in Fig. 4. As can be seen, with the increasing of feedback strength, an evaluation-route quite different from a single-mode laser is observed. The two lasing modes do not follow a period doubling or quasi-periodicity route due to the nonlinear coupling of them in the laser cavity. From the top to the bottom of Fig. 4, the feedback strength is (a) free-running; (b) −5 dBm; (c) −2.9 dBm; (d) −0.8 dBm; (e) 3.7 dBm and (f) 6 dBm, respectively. As shown in Fig. 4(b), at −5-dBm feedback strength, some side peaks around the two main modes appear in optical spectrum and the corresponding beating RF signal broadens. These side modes result from feedback-induced undamped relaxation oscillation^27^. The broadening of the beating signal is caused by the linewidth broadening of the two lasing modes under optical feedback^28^. With the increasing of feedback strength, more side peaks are excited as shown in Fig. 4(c). In the RF spectrum, the beating signal broadens continuously. And the frequency components around the relaxation frequency (≈10 *GHz*) can be observed. When the feedback strength is −0.8 dBm, as shown in Fig. 4(d), frequency components around the relaxation frequency enhances dramatically. Due to the nonlinear mixing between the beating frequency components and relaxation frequency components, some new frequencies around 18 GHz appear. Through further increasing of the feedback strength, those frequency peaks broaden gradually and connect with each other due to the enhanced lower frequency components and wave-mixing effect, as shown in Fig. 4(e). When the feedback strength reaches 6 dBm, both the optical and RF spectra expand and flat significantly as shown in Fig. 4(f). A flat chaos spectrum with wide frequency range is achieved. The chaotic source maintains this state until the feedback strength increases to 10 dBm. Further increasing of feedback power is not performed to prevent possible damage to the device.

### Post data processing

Due to the residual correlation and biases of the chaotic source, post data processing is necessary for extracting random bits from the generated chaotic waveform. Enhancing the quality of the chaotic source can efficiently simplify this procedure and correspondingly simplify the electronic back ends when implemented in real applications.

**Figure 5:**
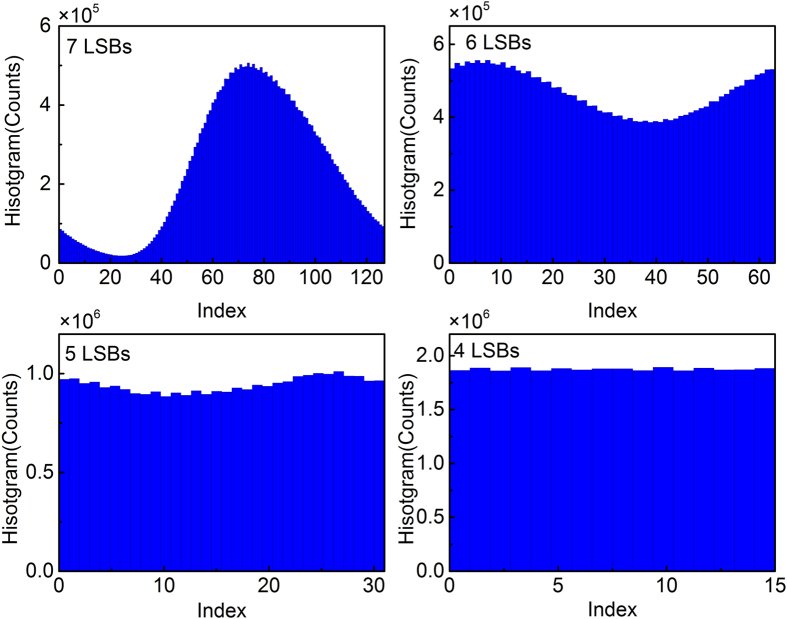
Probability distribution histogram of the digitalized chaotic waveform with (**a**) 7-LSBs, (**b**) 6-LSBs, (**c**) 5-LSBs and (**d**) 4-LSBs retained from each 8-bit sample.

**Figure 6:**
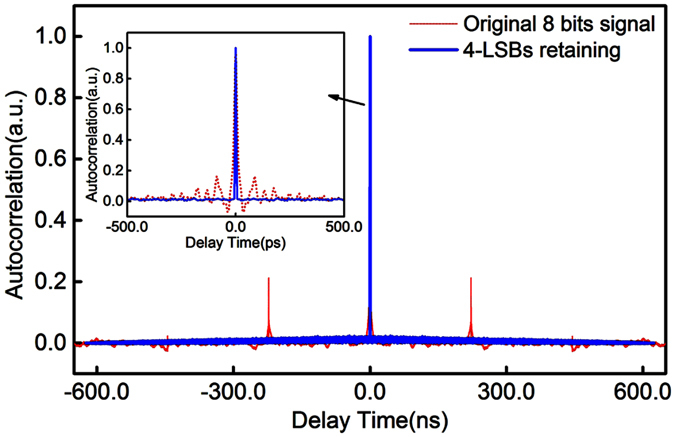
Autocorrelation functions for 4-LSBs retaining signal (blue line) and original 8-bits signal (red dots). Inset: zoom in view of the center correlation peak.

Benefited from the quality of our chaotic source, only a simple and efficient LSBs post data processing method is applied to our experiments. The most significant bits (MSBs) in a binary number are the bits at positions of the higher exponents of 2. They represent the overall fluctuation and bias of the sampling point, which contain the information of residual correlations. Therefore, eliminating several MSBs and retaining a number of LSBs can destroy the residual correlations and efficiently improve the uniformity of the bit distributions. Figure 5(a–d) illustrate the distributions of 30 million bits when different numbers of LSBs are retained. Figure 5(a) is the histogram for the 7-LSBs signal, which shows an obvious non-uniform distribution that certain values have higher probabilities. Through more numbers of MSBs truncation, as shown in Fig. 5(b,c) where 6- and 5-LSBs are retained, respectively, the uniformity of the bits distribution is clearly improved. When 4-LSBs of each 8-bit sample are retained, a flat histogram is achieved as shown in Fig. 5(d). Furthermore, the effects of LSBs on the elimination of residual correlations are investigated. Figure 6 shows the autocorrelation function of the chaotic signal under 4-LSBs retaining. To make a comparison, the autocorrelation function of the original 8-bits signal (Fig. 3(c)) is also placed in Fig. 6. As can be seen, the autocorrelation peaks corresponding to the feedback time and the side lobes around the main peak are removed when 4 MSBs are eliminated. Thus, retaining of 4 LSBs are sufficient to provide a flat histogram and eliminate the residual correlations that appear in the chaotic waveform.

### Ultra-fast random number generation

**Figure 7:**
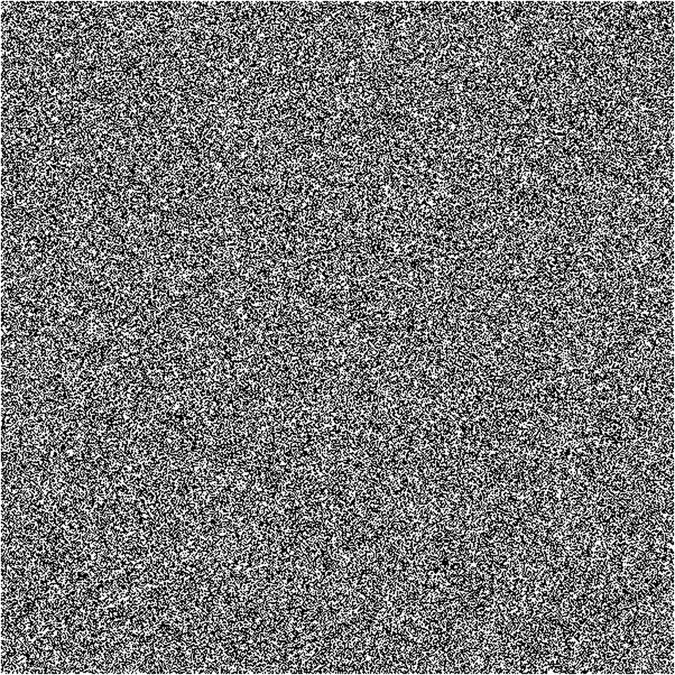
Random number patterns with 600 × 600 dots in two-dimensional plane. Bits “0” and “1” are converted to white and black dots, respectively.

In random bits generation procedure, the wideband digital oscilloscope recorded 1 Gbit samples of 8-bit data at 160 GS/s sampling rate. Those samples were truncated from the initial signal to its 4 LSBs. Then, random sequences with a rate up to 640 Gbit/s (160 *GS/s* × 4 *bits*) were generated. Figure 7 shows a visualization of the randomness of the generated bits. 600 × 600 black and white dots, which represents ‘1’ and ‘0’, are placed from left to right and from top to bottom. As can be seen, there are no obvious patterns in the figure which indicates a good randomness. To verify the statistical randomness of the generated random bits, the recorded 1 Gbit data were divided into 1000 sequences of 1 Mbit data and evaluated using standard test provided by National Institute of Standards and Technology (NIST SP 800-22). As show in Table 1, the generated random bits successfully pass all the 15 NIST tests.

## Conclusion

We experimentally demonstrated a simple scheme to generate fast physical random bits up to 640 Gbit/s. The flat broadband chaotic source was realized by a monolithically integrated dual-mode AFL with self-injection, which achieved an RF frequency range of above 50 GHz and a flatness of ±3.6 dB. The dynamic routes from dual-mode lasing into the chaotic state were discussed in detail. By using 4-least significant bits (LSBs) truncation from 8-bit digitization at 160 GS/s, high-quality physical random bits were successfully generated. This fast random number generator is simple in both chaotic source and data processing, which has great potential to be used in practical systems.

## Additional Information

**How to cite this article:** Zhang, L. *et al*. 640-Gbit/s fast physical random number generation using a broadband chaotic semiconductor laser. *Sci. Rep.* **7**, 45900; doi: 10.1038/srep45900 (2017).

**Publisher's note:** Springer Nature remains neutral with regard to jurisdictional claims in published maps and institutional affiliations.

## Figures and Tables

**Table 1:** Results of statistical test suite NIST SP800-22 for a set of 1000 sequences of 1 Mbit each.

Statistical Test	P-Value	Proportion	Result
Frequency	0.0078	0.989	Success
Block frequency	0.1134	0.997	Success
Runs	0.4637	0.984	Success
Longest run	0.7702	0.992	Success
Rank	0.7399	0.994	Success
FFT	0.6397	0.992	Success
Nonoverlapping template	0.0149	0.984	Success
Overlappng template	0.1684	0.989	Success
Universal	0.0263	0.989	Success
Linear complexity	0.8409	0.992	Success
Serial	0.2774	0.982	Success
Approximate entropy	0.4588	0.992	Success
Cumulative sums	0.3588	0.987	Success
Random excursions	0.0208	0.982	Success
Random excursions variant	0.0454	0.992	Success
